# Bioinformatic identification of proteins with tissue-specific expression for biomarker discovery

**DOI:** 10.1186/1741-7015-10-39

**Published:** 2012-04-19

**Authors:** Ioannis Prassas, Caitlin C Chrystoja, Shalini Makawita, Eleftherios P Diamandis

**Affiliations:** 1 Department of Laboratory Medicine and Pathobiology, University of Toronto, Toronto, ON, Canada; 2 Department of Clinical Biochemistry, University Health Network, Toronto, ON, Canada; 3 Department of Pathology and Laboratory Medicine, Mount Sinai Hospital, Toronto, ON, Canada

**Keywords:** bioinformatics, biomarkers, tissue-specific proteins

## Abstract

**Background:**

There is an important need for the identification of novel serological biomarkers for the early detection of cancer. Current biomarkers suffer from a lack of tissue specificity, rendering them vulnerable to non-disease-specific increases. The present study details a strategy to rapidly identify tissue-specific proteins using bioinformatics.

**Methods:**

Previous studies have focused on either gene or protein expression databases for the identification of candidates. We developed a strategy that mines six publicly available gene and protein databases for tissue-specific proteins, selects proteins likely to enter the circulation, and integrates proteomic datasets enriched for the cancer secretome to prioritize candidates for further verification and validation studies.

**Results:**

Using colon, lung, pancreatic and prostate cancer as case examples, we identified 48 candidate tissue-specific biomarkers, of which 14 have been previously studied as biomarkers of cancer or benign disease. Twenty-six candidate biomarkers for these four cancer types are proposed.

**Conclusions:**

We present a novel strategy using bioinformatics to identify tissue-specific proteins that are potential cancer serum biomarkers. Investigation of the 26 candidates in disease states of the organs is warranted.

## Background

Serological biomarkers represent a non-invasive and cost-effective aid in the clinical management of cancer patients, particularly in areas of disease detection, prognosis, monitoring and therapeutic stratification. For a serological biomarker to be useful for early detection, its presence in serum must be relatively low in healthy individuals and those with benign disease. The marker must be produced by the tumor or its microenvironment and enter the circulation, giving rise to increased serum levels. Mechanisms that facilitate entry to the circulation include secretion or shedding, angiogenesis, invasion and destruction of tissue architecture [1]. The biomarker should preferably be tissue specific, such that a change in serum level can be directly attributed to disease (for example, cancer) of that tissue [2]. The currently most widely used serological biomarkers include carcinoembryonic antigen (CEA) and carbohydrate antigen 19.9 for gastrointestinal cancer [3-5]; CEA, cytokeratin 19 fragment, neuron-specific enolase, tissue polypeptide antigen, progastrin-releasing peptide and squamous cell carcinoma antigen for lung cancer [6]; CA 125 for ovarian cancer [2]; and prostate-specific antigen (PSA, also known as kallikrein-related peptidase (KLK) 3) in prostate cancer [7]. These current serological biomarkers lack the appropriate sensitivity and specificity to be suitable for early cancer detection.

Serum PSA is commonly used for prostate cancer screening in men over 50 years old, but its usage remains controversial due to serum elevation in benign disease as well as prostate cancer [8]. Nevertheless, PSA represents one of the most useful serological markers currently available. PSA is strongly expressed only in the prostate tissue of healthy men, with low levels in the serum established by normal diffusion through various anatomical barriers. These anatomical barriers are disrupted upon development of prostate cancer, allowing increased amounts of PSA to enter circulation [1].

Recent advances in high-throughput technologies (for example, high-content microarray chips, serial analysis of gene expression, expressed sequence tags) have enabled the creation of publicly available gene and protein databases that describe the expression of thousands of genes and proteins in multiple tissues. In this study we used five gene databases and one protein database. The C-It [9,10], Tissue-specific and Gene Expression and Regulation (TiGER) [11,12] and UniGene [13,14] databases are based on expressed sequence tags (ESTs). The BioGPS [15-17] and VeryGene [18,19] databases are based on microarray data. The Human Protein Atlas (HPA) [20,21] is based on immunohistochemistry (IHC) data.

Our laboratory has previously characterized the proteomes of conditioned media (CM) from 44 cancer cell lines, three near normal cell lines and 11 relevant biological fluids (for example, pancreatic juice and ascites) using multidimensional liquid chromatography tandem mass spectrometry, identifying between 1,000 and 4,000 proteins per cancer site [22-33] (unpublished work).

Numerous candidate biomarkers have been identified from *in silico *mining of gene-expression profiling [34-36] and the HPA [37-48]. In the present study, we describe a strategy to identify tissue-specific proteins using publicly available gene and protein databases. Our strategy mines databases for proteins highly specific to or strongly expressed in one tissue, selects proteins which are secreted or shed, and integrates proteomic datasets enriched for the cancer secretome to prioritize candidates for further verification and validation studies. Integrating and comparing proteins identified from databases based on different data sources (ESTs, microarray and IHC) with the proteomes of the CM of cancer cell lines and relevant biological fluids will minimize the shortcomings of any one source, resulting in the identification of more promising candidates. Recently, the value of using an integrated approach in biomarker discovery has been described [49].

In this study, we looked at identifying tissue-specific proteins as candidate biomarkers for colon, lung, pancreatic and prostate cancer. Our strategy can be applied to identify tissue-specific proteins for other cancer sites. Colon, lung, pancreatic and prostate cancer are ranked among the top leading causes of cancer-related deaths, cumulatively accounting for an estimated half of all cancer-related deaths [50]. Early diagnosis is essential for improving patient outcomes as early-stage cancers are less likely to have metastasized and are more amenable to curative treatment. The five-year survival rate when treatment is administered on metastatic stages compared to organ-confined cancer drops dramatically from 91% to 11% in colorectal cancer, 53% to 4% in lung cancer, 22% to 2% in pancreatic cancer and 100% to 31% in prostate cancer [50].

We identified 48 tissue-specific proteins as candidate biomarkers for the selected tissue types. Of these, 14 had been previously studied as cancer or benign disease serum biomarkers, providing credence to our strategy. Investigation of the remaining proteins in future studies is warranted.

## Methods

### *In silico *discovery

Six gene and protein databases were mined to identify proteins highly specific to or strongly expressed in one tissue. Colon, lung, pancreatic and prostate tissues were examined.

The C-It database [10] was searched for each tissue for proteins enriched in that selected tissue (human data only). Since the C-It database did not have colon data available, only lung, pancreatic and prostate tissue were searched. Literature information search parameters of fewer than five publications in PubMed and fewer than three publications with the Medical Subject Headings (MeSH) term of the searched tissue were used. The option of adding z-scores of the corresponding SymAtlas microarray probe sets to the protein list was included [16]. Only proteins with a corresponding SymAtlas z-score of ≥|1.96|, corresponding to a 95% confidence level of enrichment, were included in our lists. Proteins without a SymAtlas z-score were ignored. The TiGER database [12] was searched for proteins preferentially expressed in each tissue based on ESTs by searching each tissue using 'Tissue View'. The UniGene database [14] was searched for tissue-restricted genes using the following search criteria: [tissue][restricted] + "*Homo sapiens*", for the lung, pancreatic and prostate tissues. Since the UniGene database did not have data for colon tissue, a search of: [colorectal tumor][restricted] + "*Homo sapiens*" was used.

The BioGPS database (v. 2.0.4.9037; [17]) plugin 'Gene expression/activity chart' using the default human data set 'GeneAtlas U133A, gcrma' [16] was searched with a protein whose gene expression profile using the BioGPS plugin showed it to be specific to and strongly expressed in one tissue of interest. Chloride channel accessory 4, surfactant protein A2, pancreatic lipase (PNLIP) and KLK3 were selected for colon, lung, pancreatic and prostate tissues, respectively. For each protein searched, a correlation cutoff of 0.9 was used to generate a list of proteins with a similar expression pattern to the initial protein searched. Each tissue was searched in the VeryGene database [19] using 'Tissue View' for tissue-selective proteins.

The HPA [21] was searched for proteins strongly expressed in each normal tissue with annotated expression. Annotated protein expression is a manually curated score based on IHC staining patterns in normal tissues from two or more paired antibodies binding to different epitopes of the same protein, which describes the distribution and strength of expression of each protein in cells [51].

### Identification of protein overlap in databases

An in-house developed Microsoft Excel macro was utilized to evaluate the number of times a protein was identified in each tissue and which database had identified it. Proteins identified in only one database were eliminated. Proteins identified in two or more databases could represent candidates that are more promising at this stage, since databases based on varying sources of data identified the protein as being highly specific to or strongly expressed in one tissue.

### Secreted or shed proteins

For each tissue type, the list of proteins identified in two or more databases was exported into a comma-delimited Microsoft Excel file. An in-house secretome algorithm (GS Karagiannis *et al.*, unpublished work) was applied to identify proteins that are either secreted or shed. The secretome algorithm designates a protein as secreted or shed if it is either predicted to be secreted based on the presence of a signal peptide or through non-classical secretion pathways, or predicted to be a membranous protein based on amino-acid sequences corresponding to transmembrane helices. Proteins that were not designated as secreted or shed were eliminated.

### Verification of *in silico *expression profiles

The BioGPS and HPA databases were used to manually verify the expression profiles of the proteins identified as being secreted or shed for strength and specificity of expression. The BioGPS database was chosen above the other gene databases as it offers a gene expression chart and the ability to batch search for a list of proteins, which allowed efficient searching and verification of protein lists. If expression profiles were not available in the BioGPS database, the protein was eliminated.

The BioGPS database plugin 'Gene expression/activity chart' using the default human data set 'GeneAtlas U133A, gcrma' was searched for each protein. For each tissue, proteins with gene expression profiles showing similar values of expression or strong expression in more than the selected tissue were eliminated (strong expression is defined as ≥ 10 times the median expression value in all tissues). In BioGPS, the color of the bars in the 'Gene expression/activity chart' reflects a grouping of similar samples, based on global hierarchical clustering. If strong expression was seen in more than the selected tissue, but only in tissues with the same bar color, the protein was not eliminated.

The HPA was searched for each protein, and the 'Normal Tissue' expression page was evaluated. Tissue presentation order by organ was selected. An evaluation of the protein's expression in normal tissue was preferably based on the level of annotated protein expression or, if the annotated expression was not available, the level of antibody staining. The levels of annotated protein expression are none, low, medium and high and the levels of antibody staining are negative, weak, moderate and strong. For each tissue, proteins with high/strong expression in the selected tissue and medium/moderate expression in more than two other tissues were eliminated. Proteins with high/strong or medium/moderate expression in more than the one selected tissue were eliminated. Proteins with low/weak or none/negative expression in the selected tissue were eliminated. If the high/strong or the medium/moderate level was seen in more than the one selected tissue, where the other tissues were in the same organ, and low/weak or none/negative expression was seen in all other tissues, the protein was included.

Proteins with pending HPA data were evaluated based on their gene expression profiles. Proteins were also eliminated when their HPA protein expression profiles fit the criteria for elimination but their gene expression profiles did not fit the criteria for elimination.

### Literature search

The PubMed database was manually searched for each of the proteins whose expression profile was verified *in silico*. For each tissue, proteins that had been previously studied as candidate cancer or benign disease serum biomarkers in the selected tissue were eliminated. Proteins with high abundance in serum ( > 5 μg/mL) or known physiology and expression were also eliminated.

### Proteomic datasets

An in-house Microsoft Excel macro was utilized for comparison of the remaining protein lists against previously characterized in-house proteomes of the CM from 44 cancer cell lines, three near normal cell lines and 11 relevant biological fluids [22-33] (unpublished work). Proteomes were characterized using multidimensional liquid chromatography tandem mass spectrometry on a linear ion trap (LTQ) Orbitrap mass spectrometer (Thermo Fisher Corporation, Pittsburgh, PA, USA). For details, see our previous publications [22-33]. The cancer cell lines were from six cancer types (breast, colon, lung, ovarian, pancreatic and prostate). The relevant biological fluids included amniotic fluid (normal, with Down Syndrome), nipple aspirate fluid, non-malignant peritoneal fluid, ovarian ascites, pancreatic ascites, pancreatic juice, pancreatic tissue (normal and malignant) and seminal plasma. A complete list of cell lines and relevant biological fluids is provided in Additional file 1. If a protein was identified in amniotic fluid and the proteome of a tissue, this was noted but not considered as expression in a non-tissue proteome.

The data of proteomes from the CM of 23 cancer cell lines (from 11 cancer types), as recently published by Wu *et al. *[52], was also integrated. Proteomes were characterized using one-dimensional SDS-PAGE and nano-liquid chromatography tandem mass spectrometry on a LTQ-Orbitrap mass spectrometer. The 11 cancer types included breast, bladder, cervical, colorectal, epidermoid, liver, lung, nasopharyngeal, oral and pancreatic cancer, and T-cell lymphoma [52]. If a protein was identified in a proteomic dataset, the proteome in which it was identified was noted.

**Figure 1:**
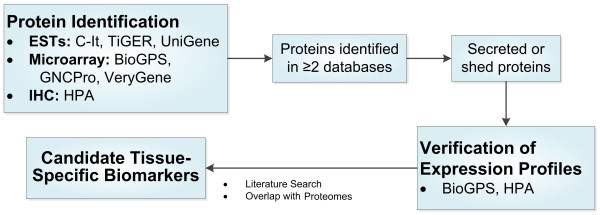
**Schematic outline of tissue-specific biomarker identification**. Protein identification in six publicly available gene and protein databases, grouped by the type of data each database is based on, followed by filtering criteria and integration of proteomic datasets to identify and prioritize candidates is outlined. ESTs: expressed sequence tags; HPA: Human Protein Atlas; IHC: immunohistochemistry; TiGER: Tissue-specific and Gene Expression and Regulation.

A schematic outline of the methodology is provided in Figure 1.

## Results

### Identification of proteins

A total of 3,615 proteins highly specific to or strongly expressed in the colon, lung, pancreas or prostate were identified in the databases. Searching the databases identified 976 unique proteins that were highly specific to or strongly expressed in the colon, 679 for the lung, 1,059 for the pancreas and 623 for the prostate (Table 1). For the four tissue types, the C-It database identified 254 tissue-enriched proteins, the TiGER database identified 636 proteins preferentially expressed in tissue and the UniGene database identified 84 tissue-restricted proteins. The BioGPS database identified 127 proteins similarly expressed as a protein with known tissue specificity, and the VeryGene database identified 365 tissue-selective proteins. The HPA identified 2,149 proteins showing strong tissue staining and with annotated expression. The total number of proteins identified by each database in the four tissue types contains some proteins that were identified in more than one tissue. A complete list of proteins identified in each tissue by each database is presented in Additional file 2 and is summarized in Additional file 3.

**Table 1:** Total number of proteins identified from mining gene and protein databases

	Tissue			
	Colon	Lung	Pancreas	Prostate
Total unique proteins	976	679	1059	623
[in ≥ two databases]	[32]	[36]	[81]	[48]
Number of proteins identified in				
One database	944	643	968	575
Two databases	23	30	46	32
Three databases	7	5	23	11
Four databases	1	1	9	4
Five databases	1	-	3	1
Number [%] of secreted or shed proteins in ≥ two databases^a^	26	25	58	34
	[81]	[69]	[72]	[71]

^a^Pertains to proteins identified using a secretome algorithm

### Protein identification overlap in databases

A total of 32 proteins in the colon, 36 proteins in the lung, 81 proteins in the pancreas and 48 proteins in the prostate were identified in two or more databases. Selecting for proteins identified in two or more databases eliminated between 92% and 97% of the proteins in each of the tissue types. The majority of the remaining proteins were identified in only two of the databases, and no proteins were identified in all the databases. This data is summarized in Table 1 and a complete list of proteins identified in one or more databases, including the number of databases it was identified in and which databases those were, is presented in Additional file 4 for each tissue.

### Secreted or shed proteins

The majority of the proteins identified in two or more databases were identified as being secreted or shed. In total, 143 of the 197 proteins from all tissues were designated as being secreted or shed (Table 1). Specifically, 26 proteins in the colon, 25 proteins in the lung, 58 proteins in the pancreas and 34 proteins in the prostate were designated as being secreted or shed. A complete list is provided in Additional file 5.

### Verification of *in silico *expression profiles

Manual verification of the expression profiles of the secreted or shed proteins identified in two or more databases eliminated the majority of the proteins: 21 in the colon, 16 in the lung, 32 in the pancreas and 26 in the prostate. Only five (0.5%) of the 976 proteins initially identified as highly specific to or strongly expressed in the colon were found to meet the filtering criteria. Nine (1.3%) of 679 proteins in the lung, 26 (2.4%) of 1,059 proteins in the pancreas and eight (1.3%) of 623 proteins in the prostate were found to meet the filtering criteria. These remaining 48 proteins are tissue-specific and secreted or shed and, therefore, represent candidate biomarkers (Table 2).

**Table 2:** Forty-eight proteins identified as tissue-specific, strongly expressed and secreted or shed in colon, lung, pancreatic or prostate tissue^a^

Tissue	Gene	**BioGPS **[15,16]	**C-It **[9]	**HPA **[20]	**TiGER **[11]	**UniGene **[13]	**VeryGene **[18]	Previously studied as a (tissue) cancer or benign disease serum biomarker [reference]
**Colon**	*CEACAM7*	√			√			
	*CLCA1*	√			√	√		
	*GPA33*			√	√			
	*LEFTY1*	√			√			
	*ZG16*	√			√			
**Lung**	*IRX5*	√	√		√			
	*LAMP3*				√		√	
	*MFAP4*	√	√					
	*SCGB1A1*	√			√		√	
	*SFTPA2*	√					√	[54-56]
	*SFTPB*	√			√		√	[55]
	*SFTPC*			√	√			
	*SFTPD*	√			√		√	[56]
	*TMEM100*	√	√				√	
**Pancreas**	*AQP8*	√					√	
	*CEL*	√			√		√	[60]
	*CELA2A*					√	√	[61]
	*CELA2B*	√	√			√	√	
	*CELA3B*	√				√		
	*CPA1*	√		√	√	√	√	[62]
	*CPA2*	√		√	√		√	[62]
	*CPB1*	√			√	√	√	[63]
	*CTRB1*	√			√			
	*CTRB2*	√	√			√		
	*CTRC*	√			√		√	
	*CUZD1*		√		√		√	
	*GCG*				√	√	√	
	*IAPP*				√	√	√	
	*INS*			√	√		√	
	*KLK1*	√			√		√	
	*PNLIP*	√			√	√	√	[64]
	*PNLIPRP1*		√		√		√	
	*PNLIPRP2*	√			√		√	
	*PPY*				√	√	√	
	*PRSS1*	√		√	√	√	√	[65]
	*PRSS3*				√		√	
	*REG1B*				√	√	√	
	*REG3G*		√		√		√	
	*SLC30A8*				√	√	√	
	*SYCN*	√	√		√	√	√	[33]
**Prostate**	*ACPP*	√		√	√	√	√	[66]
	*FOLH1*	√			√			[67]
	*KLK2*	√			√			[68]
	*KLK3*	√		√	√			[66]
	*NPY*				√		√	
	*PSCA*	√			√			
	*RLN1*	√			√	√	√	
	*SLC45A3*	√		√	√		√	

^a^Tissue-specific proteins as it applies to this table indicates protein expression was manually verified in BioGPS and/or HPA databases. HPA: Human Protein Atlas; TiGER: Tissue-specific and Gene Expression and Regulation.

### Performance of databases

**Figure 2:**
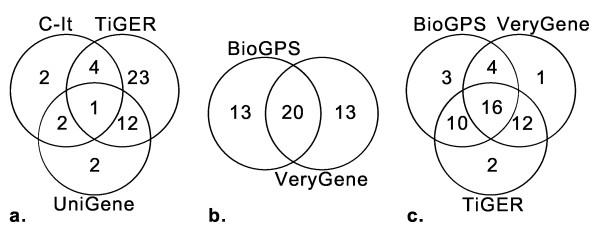
**Identification of tissue-specific proteins by each database**. Venn diagrams depicting which database had initially identified the tissue-specific proteins that passed the filtering criteria (identified in two or more databases, designated as secreted or shed, and expression profiles verified *in silico*). Overlap of tissue-specific proteins identified in databases based off **(a) **ESTs, **(b) **microarray and **(c) **three databases that identified the most tissue-specific proteins is also depicted. For details see text.

The performance of the databases was evaluated by determining how many of the 48 proteins that passed the filtering criteria were initially identified by each database (Figure 2). The TiGER database had been responsible for initially identifying the greatest number of proteins that passed the filtering criteria. The TiGER database, the BioGPS database and the VeryGene database had each identified > 68% of the 48 proteins. The TiGER database had identified 40 of the 48 proteins, and the BioGPS and VeryGene databases had both identified 33 of 48 proteins. The UniGene database identified 35% (17 out of 48) of the proteins and the C-It database and the HPA both identified 19% (9 out of 48) of the proteins (Table 2).

The accuracy of the initial protein identifications was evaluated by comparing the proportion of proteins that had passed the filtering criteria that each database had initially identified to the total number of proteins each database initially identified. The BioGPS database showed the highest accuracy of initial protein identification. Of the proteins initially identified by the BioGPS database, 26% (33 of 127) met all the filtering criteria. The UniGene database showed 20% accuracy (17 of 84), VeryGene showed 9% (33 of 365), TiGER showed 6% (40 of 636), C-It showed 4% (9 of 254) and HPA showed 0.4% (9 of 2,149).

### Literature search

None of the colon-specific proteins had been previously studied as serum colon cancer biomarkers. Surfactant proteins have been extensively studied in relation to various lung diseases [53], and surfactant protein A2, surfactant protein B and surfactant protein D have been studied as serum lung cancer or lung disease biomarkers [54-56]. Elastase proteins have been studied in pancreatic function and disease [57], islet amyloid polypeptide and pancreatic polypeptide are normally secreted [58,59], and glucagon and insulin are involved in the normal function of healthy individuals. Eight of the pancreas-specific proteins had been previously studied as serum pancreatic cancer or pancreatitis biomarkers [33,60-65]. Four of the prostate-specific proteins had been previously studied as serum prostate cancer biomarkers [66-68] (Table 2).

### Protein overlap with proteomic datasets

Of the tissue-specific proteins that had not been studied as serum tissue cancer biomarkers, 18 of the 26 proteins were identified in proteomic datasets (Tables 3, 4, 5 and 6). Nine proteins were exclusively identified in datasets of corresponding tissues. Of the colon-specific proteins, only glycoprotein A33 (GPA33) was identified exclusively in colon datasets. GPA33 was identified in the CM of three colon cancer cell lines, LS174T, LS180 and Colo205 [52] (GS Karagiannis *et al.*, unpublished work) (Table 3). None of the lung-specific proteins were identified in lung datasets (Table 4). Seven pancreas-specific proteins were exclusively identified in pancreatic datasets: in pancreatic cancer ascites [32], pancreatic juice [33] and normal or cancerous pancreatic tissue (H Kosanam *et al.*, unpublished work) (Table 5). None were identified in the CM of pancreatic cancer cell lines. Neuropeptide Y (NPY) was the only prostate-specific protein identified exclusively in prostate datasets. NPY was identified in the CM of the prostate cancer cell line VCaP (P Saraon *et al.*, unpublished work) and the seminal plasma proteome [25].

**Table 3:** List of colon tissue-specific proteins which have not been previously studied as serum cancer or benign disease biomarkers

Gene	Protein name	Proteome identified in:	
		CM proteome from colon cancer cell lines	Non-colon proteome
***CEACAM7***	Carcinoembryonic antigen-related cell adhesion molecule 7		√ CM proteome from Hep 3B [52]; pancreatic juice proteome [33]
***CLCA1***	Chloride channel accessory 1		√ Normal, Down Syndrome amniotic fluid [22,23]
***GPA33***	Glycoprotein A33	√ LS174T^a^, LS180^a^, Colo205 [52]	
***LEFTY1***	Left-right determination factor 1		
***ZG16***	Zymogen granule protein 16 homolog (rat)		√ CM proteome from Hep 3B [52]

^a^CM proteome of colon cancer cell lines (GS Karagiannis *et al.*, unpublished work). CM: conditioned media.

**Table 4:** List of lung tissue-specific proteins which have not been previously studied as serum cancer or benign disease biomarkers

Gene	Protein name	Proteome identified in:	
		**CM proteome from lung cancer cell lines **[27,52]	Non-lung proteome
***IRX5***	Iroquois homeobox 5		
***LAMP3***	Lysosomal-associated membrane protein 3		
***MFAP4***	Microfibrillar-associated protein 4		√ Normal and cancer pancreatic tissue^a^, seminal plasma proteome [25]; non-malignant peritoneal fluid [26]
***SCGB1A1***	Secretoglobin, family 1A, member 1 (uteroglobin)		√ [22,23,25,26,31-33]
***TMEM100***	Transmembrane protein 100		

^a^Proteome of normal and cancer pancreatic tissue (H Kosanam *et al.*, unpublished work). CM: conditioned media.

**Table 5:** List of pancreas tissue-specific proteins which have not been previously studied as serum cancer or benign disease biomarkers

Gene	Protein name	Proteome identified in:					
		**CM proteome from pancreatic cancer cell lines **[33]	**Pancreatic cancer ascites proteome **[32]	**Pancreatic juice proteome **[33]	Pancreatic tissue^a^		Non-pancreas proteome
					Normal	Cancer	
***AQP8***	Aquaporin 8						
***CTRB1***	Chymotrypsinogen B1		√				√ Down Syndrome amniotic fluid [22]
***CTRB2***	Chymotrypsinogen B2			√	√		
***CTRC***	Chymotrypsin C (caldecrin)			√	√	√	
***CUZD1***	CUB and zona pellucida-like domains 1			√	√		
***KLK1***	Kallikrein 1			√	√	√	
***PNLIPRP1***	Pancreatic lipase-related protein 1			√	√		
***PNLIPRP2***	Pancreatic lipase-related protein 2			√	√		√ CM proteome from Hep 3B [52]
***PRSS3***	Protease, serine, 3		√	√	√	√	√ HCC-38^b^; HCC-1143^b^; normal amniotic fluid [23]
***REG1B***	Regenerating islet-derived 1 beta			√	√	√	
***REG3G***	Regenerating islet-derived 3 gamma			√			√ Seminal plasma proteome [25]
***SLC30A8***	Solute carrier family 30 (zinc transporter), member 8						

^a^Proteome of normal and cancer pancreatic tissue (H Kosanam *et al.*, unpublished work); ^b^CM proteome of breast cancer cell lines (M Pavlou *et al.*, unpublished work). CM: conditioned media.

**Table 6:** List of prostate-specific proteins which have not been previously studied as serum cancer or benign disease biomarkers

**Gene**	**Protein name**	**Proteome identified in:**		
		**CM proteome from prostate cancer cell lines**	**Seminal plasma proteome**[25]	**Non-prostate proteome**
***NPY***	Neuropeptide Y	√ VCaP^a^	√	
***PSCA***	Prostate stem cell antigen	√ PC3 [28]	√	√ Normal and cancer pancreatic tissue^b^; CM proteome from pancreatic cancer cell lines SU.86.86, CAPAN1 [33]
***RLN1***	Relaxin 1			
***SLC45A3***	Solute carrier family 45, member 3			

^a^CM proteome from prostate cancer cell line (P Saraon *et al.*, unpublished work); ^b^proteome of normal and cancer pancreatic tissue (H Kosanam *et al.*, unpublished work). CM: conditioned media.

## Discussion

We describe a strategy to identify tissue-specific biomarkers using publicly available gene and protein databases. Since serological biomarkers are protein-based, using only protein expression databases for the initial identification of candidate biomarkers seems more relevant. While the HPA has characterized more than 50% of human protein-encoding genes (11,200 unique proteins to date), it has not completely characterized the proteome [51]. Therefore, proteins that have not been characterized by the HPA but fulfill our desired criteria would be missed by searching only the HPA. There are also important limitations in using gene expression databases since there is considerable variation between mRNA and protein expression [69,70] and gene expression does not account for post-translational modification events [71]. Therefore, mining both gene and protein expression databases minimizes the limitations of each platform. To the best of our knowledge, no studies for the initial identification of candidate cancer biomarkers have been conducted using both gene and protein databases.

Initially, the databases were searched for proteins highly specific to or strongly expressed in one tissue. The search criteria were tailored to accommodate the design of the databases, which did not allow for simultaneous searching with both criteria. Identifying proteins that were highly specific to and strongly expressed in one tissue was considered in a later step. In the verification of the expression profiles (see *Methods*), only 34% (48 of 143) of the proteins were found to meet both criteria. The number of databases mined in the initial identification can be varied at the discretion of the investigator. Additional databases will result in the same number of, or more, proteins being identified in two or more databases.

In the gene expression databases, the criteria used were set for maximum stringency for protein identification, to identify a manageable number of candidates. A more exhaustive search can be conducted using lower stringency criteria. The stringency could be varied in the correlation analysis using the BioGPS database plugin and the C-It database. The correlation cutoff of 0.9 used in identifying similarly expressed genes in the BioGPS database plugin could be reduced to as low as 0.75. The SymAtlas z-score of ≥|1.96| could be reduced to ≥|1.15|, corresponding to a 75% confidence level of enrichment. The literature information parameters used in the C-It database of fewer than five publications in PubMed and fewer than three publications with the MeSH term of the selected tissue could be reduced in stringency, to allow identification of well-studied proteins. Since C-It does not look at the content of publications in PubMed, it filters out proteins that have been studied even if they have not been studied in relation to cancer.

Although proteins that have been well studied but not as cancer biomarkers represent potential candidates, the emphasis in this study was on identifying novel candidates which have been, overall, minimally studied. A gene's mRNA level and protein expression can have significant variability. Therefore, if lower stringency criteria were used when identifying proteins from gene expression databases, a greater number of proteins would have been identified in at least two of the databases, potentially leading to a greater number of candidate protein biomarkers identified after application of the remaining filtering criteria.

The HPA was searched for proteins strongly expressed in one normal tissue with annotated IHC expression. Annotated IHC expression was selected because it uses paired antibodies to validate the staining pattern, providing the most reliable estimation of protein expression. Approximately 2,020 of the 10,100 proteins in version 7.0 of the HPA have annotated protein expression [51]. Makawita *et al. *[33] included the criteria of annotated protein expression when searching for proteins with 'strong' pancreatic exocrine cell staining for prioritization of pancreatic cancer biomarkers. A more exhaustive search could be conducted by searching the HPA without annotated IHC expression.

Secreted or shed proteins have the highest chance of entering the circulation and being detected in the serum. Many groups, including ours [23-25,27-33], use Gene Ontology [72] protein cellular localization annotations of 'extracellular space' and 'plasma membrane' to identify a protein as secreted or shed. Gene Ontology cellular annotations do not completely describe all proteins and are not always consistent if a protein is secreted or shed. An in-house secretome algorithm (GS Karagiannis *et al.*, unpublished work) designates a protein as secreted or shed if it is predicted either to be secreted based on the presence of signal peptide or to have non-classical secretion, or predicted to be a membranous protein based on amino-acid sequences corresponding to transmembrane helices. It more robustly defines proteins as secreted or shed and was therefore used in this study.

Evaluating which of the databases had initially identified the 48 tissue-specific proteins that passed the filtering criteria showed that the gene expression databases had identified more of the proteins than the protein expression database. The HPA had initially identified only 9 of the 48 tissue-specific proteins. The low initial identification of tissue-specific proteins was due to the stringent search criteria requiring annotated IHC expression. For example, 20 of the 48 tissue-specific proteins had protein expression data available in the HPA, of which the 11 proteins that were not initially identified by HPA did not have annotated IHC expression. The expression profiles of those proteins would have passed the 'Verification of *in silico *expression profiles' filtering criteria and, therefore, would have resulted in a greater initial identification of tissue-specific proteins by the HPA.

The HPA has characterized 11,200 unique proteins, which is more than 50% of the human protein-encoding genes [51]. Of the 48 tissue-specific proteins that met the selection criteria, only nine were initially identified from mining the HPA. Twenty of the tissue-specific proteins have been characterized by the HPA. This demonstrates the importance of combining gene and protein databases to identify candidate cancer serum biomarkers. If only the HPA had been searched for tissue-specific proteins, even with lowered stringency, the 28 proteins that met the filtering criteria and represent candidate biomarkers would not have been identified.

The TiGER, UniGene and C-It databases are based on ESTs and collectively identified 46 of the 48 proteins. Of those, only 41% (19 of the 46) were identified in two or more of those databases. The BioGPS and VeryGene databases are based on microarray data and collectively identified 46 of the 48 proteins. Of those, 56% (26 of the 46) were identified uniquely by BioGPS and VeryGene. Clearly, even though databases are based on similar sources of data, individual databases still identified unique proteins. This demonstrates the validity of our initial approach of using databases that differently mine the same data source. The TiGER, BioGPS and VeryGene databases collectively identified all 48 of the tissue-specific proteins. From those three databases, 88% (42 of the 48) were identified in two or more databases, demonstrating the validity of selecting proteins identified in more than one database.

The accuracy of the databases' initial protein identification is related to how explicitly the database could be searched for the filtering criteria of proteins highly specific to and strongly expressed in one tissue. The BioGPS database had the highest accuracy at 26%, as it was searched for proteins similarly expressed as a protein of known tissue specificity and strong expression. The UniGene database, with an accuracy of 20%, could only be searched for proteins with tissue-restricted expression, without the ability to search for proteins also with strong expression in the tissue. The VeryGene database, accuracy of 9%, was searched for tissue-selective proteins and the TiGER database, with 6% accuracy, was searched for proteins preferentially expressed in a tissue. Their lower accuracies reflect that they could not be explicitly searched for proteins highly specific to only one tissue. The C-It database, with an accuracy of 4%, searched for tissue-enriched proteins and the HPA, accuracy of 0.4%, searched for proteins with strong tissue staining. These very low accuracies reflect that the search looked for proteins with strong expression in a tissue, but could not be searched for proteins highly specific to only one tissue.

The low identification of tissue-specific proteins by the C-It database is not unexpected. Given that the literature search parameters initially used filtered out any proteins that had fewer than five publications in PubMed, regardless of whether those publications were related to cancer, C-It only identified proteins enriched in a selected tissue which have been minimally, if at all, studied. Of the nine proteins C-It initially identified from the tissue-specific list, eight of the proteins had not been previously studied as serum candidate cancer biomarkers. Syncollin (SYCN) has only very recently been shown to be elevated in the serum of pancreatic cancer patients [33]. The eight remaining proteins that C-It identified represent especially interesting candidate biomarkers because they represent proteins that fulfill the filtering criteria but have not been well studied.

A PubMed search revealed that 15 of the 48 tissue-specific proteins identified had been previously studied as serum markers of cancer or benign disease, providing credence to our approach. The most widely used biomarkers currently suffer from a lack of sensitivity and specificity due to the fact they are not tissue-specific. CEA is a widely used colon and lung cancer biomarker. It was identified by the BioGPS and TiGER databases and the HPA as highly specific to or strongly expressed in the colon, but not by any of the databases for the lung. CEA was eliminated upon evaluating the protein expression profile *in silico*, because it is not tissue specific. High levels of CEA protein expression were seen in the normal tissues of the digestive tract, such as the esophagus, small intestine, appendix, colon and rectum, as well as in bone marrow, and medium levels were seen in the tonsil, nasopharynx, lung and vagina. PSA is an established, clinically relevant biomarker for prostate cancer with demonstrated tissue specificity. PSA was identified in our strategy as a prostate-specific protein, after passing all the filtering criteria. This provides credence to our approach because we re-identified known clinical biomarkers and our strategy filtered out the biomarkers based on tissue specificity.

From the list of candidate proteins that have not been studied as serum cancer or benign disease biomarkers, 18 of the 26 proteins were identified in proteomic datasets. The proteomic datasets primarily contain the CM proteomes of various cancer cell lines, and other relevant fluids, enriched for the secretome. For proteins that have not been characterized by the HPA, it is possible the transcripts are not translated, in which case they would represent unviable candidates. If the transcripts are translated and the protein enters circulation, it must do so at a level detectable by current proteomic techniques. Proteins that have been characterized by the HPA may not necessarily enter the circulation. The identification of a protein in the proteomic datasets verifies the presence of the protein in the secretome of cancer at a detectable level; therefore, the protein represents a viable candidate. Because cancer is a highly heterogeneous disease, the integration of multiple cancer cell lines and relevant biological fluids likely provides a more, if not necessarily complete picture of the cancer proteome.

Relaxin 1 is a candidate protein that was not identified in any of the proteomes but its expression was confirmed by semi-quantitative RT-PCR in prostate carcinomas [73]. Therefore, a protein not being identified in any of the proteomic datasets does not necessarily imply that it is not expressed in cancer.

Acid phosphatase is a previously studied prostate cancer serum biomarker [74]. When compared to proteomic datasets (data not shown), it was identified in the seminal plasma proteome [25], the CM of many prostate cancer cell lines [28] (P Saraon *et al.*, unpublished work) and, interestingly, the CM of colon cancer cell lines Colo205 [52] and LS180 (GS Karagiannis *et al.*, unpublished work), the CM of breast cancer cell lines HCC-1143 (MP Pavlou *et al.*, unpublished work) and MCF-7 [52], the CM of oral cancer cell line OEC-M1 [52] and the CM of ovarian cancer cell line HTB161 (N Musrap *et al.*, unpublished work). Graddis *et al. *[74] observed very low levels of acid phosphatase mRNA expression in both normal and cancerous breast and colon tissue, in normal ovary and salivary gland tissue and comparatively high levels in normal and malignant prostate tissue. We, therefore, reasoned that identification of a tissue-specific protein in a proteome of a different tissue does not necessarily correlate with strong expression in that proteome.

Identification of a tissue-specific protein in only proteomes corresponding to that tissue, coupled with *in silico *evidence of strong and specific protein expression in that tissue, indicates an especially promising candidate cancer biomarker. SYCN has been shown to be increased in the serum of pancreatic cancer patients [33]. SYCN was identified in the pancreatic juice proteome [33] and in normal pancreatic tissue (H Kosanam *et al.*, unpublished work) and by BioGPS, C-It, TiGER, UniGene and VeryGene databases as strongly expressed in only the pancreas. Folate hydrolase 1, also known as prostate-specific membrane antigen, and KLK2 have been studied as prostate cancer serum biomarkers [67,68]. Folate hydrolase 1 and KLK2 were both identified in the CM of various prostate cancer cell lines [28] (P Saraon *et al.*, unpublished work) and the seminal plasma proteome [25] and by BioGPS and TiGER databases as strongly expressed in only the prostate. Of the tissue-specific proteins which have not been previously studied as serum cancer or benign disease biomarkers, colon-specific protein GPA33, pancreas-specific proteins chymotrypsinogen B1 and B2, chymotrypsin C, CUB and zona pellucida-like domains 1, KLK1, PNLIP-related protein 1 and 2, regenerating islet-derived 1 beta and 3 gamma and prostate-specific protein NPY represent such candidates. Investigation of these candidates should be prioritized for further verification and validation studies.

The proposed strategy seeks to identify candidate tissue-specific biomarkers for further experimental studies. Using colon, lung, pancreatic and prostate cancer as case examples, we identified a total of 26 tissue-specific candidate biomarkers. In the future, we intend to validate the candidates; if validation is successful, we can validate the use of this strategy for *in silico *cancer biomarker discovery. Using this strategy, investigators can rapidly screen for candidate tissue-specific serum biomarkers and prioritize candidates for further study based on overlap with proteomic datasets. This strategy can be used to identify candidate biomarkers for any tissue, contingent on the data availability in the mined databases, and incorporate various proteomic datasets at the discretion of the investigator.

## Conclusions

We present a novel strategy using bioinformatics to identify tissue-specific proteins that are potential cancer serum biomarkers. Investigation of the 26 candidates in disease states of the organs is warranted.

## Abbreviations

CA: carbohydrate antigen; CEA: carcinoembryonic antigen; CM: conditioned media; CYFRA 21-1: cytokeratin 19 fragment; ESTs: expressed sequence tags; GPA33: glycoprotein A33; HPA: Human Protein Atlas; IHC: immunohistochemistry; KLK: kallikrein-related peptidase; MeSH: Medical Subject Headings; NPY: neuropeptide Y; PNLIP: pancreatic lipase; PSA: prostate-specific antigen; RT-PCR: reverse transcriptase polymerase chain reaction; SYCN: syncollin; TiGER: Tissue-specific and Gene Expression and Regulation.

## Competing interests

Parts of this paper have been included in a provisional patent application, filed by The University Health Network, Toronto, Ontario, Canada.

## Authors' contributions

IP, CCC and SM conceived the study and performed all experiments. CCC and EPD interpreted the data and drafted and revised the manuscript. All authors read and approved the final manuscript.

## Pre-publication history

The pre-publication history for this paper can be accessed here:

http://www.biomedcentral.com/1741-7015/10/39/prepub

## Supplementary Material

Additional file 1 **List of in-house proteomes**. Table listing the cell lines and relevant biological fluids of previously characterized in-house proteomes. Click here for file

Additional file 2 **List of all proteins initially identified**. Table with lists of proteins identified in each database for each tissue. Click here for file

Additional file 3 **Summary of total protein identification by each database**. Table summarizing the number of proteins identified in each tissue, by each database. Click here for file

Additional file 4 **List of the databases each protein was identified in**. Table with number of databases a protein was identified in and which databases those were for proteins identified in one or more databases for each tissue. Click here for file

Additional file 5 **Proteins identified in two or more databases and secreted or shed**. Table listing the 143 proteins designated as secreted or shed from all tissues, which were identified in two or more databases. Click here for file
